# Translational research: are community-based child obesity treatment programs scalable?

**DOI:** 10.1186/s12889-015-2031-8

**Published:** 2015-07-14

**Authors:** Louise L. Hardy, Seema Mihrshahi, Joanne Gale, Binh Nguyen, Louise A. Baur, Blythe J. O’Hara

**Affiliations:** Prevention Research Collaboration, Sydney School of Public Health, University of Sydney, Sydney, NSW 2006 Australia; Discipline of Paediatrics and Child Health, The Children’s Hospital at Westmead Clinical School, Westmead, NSW Australia

**Keywords:** Community health services, Behaviour change, Public health

## Abstract

**Background:**

Community-based obesity treatment programs have become an important response to address child obesity; however the majority of these programs are small, efficacy trials, few are translated into real-world situations (i.e., dissemination trials). Here we report the short-term impact of a scaled-up, community-based obesity treatment program on children’s weight and weight-related behaviours disseminated under real world conditions.

**Methods:**

Children age 6–15 years with a body mass index (BMI) ≥85th percentile with no co-morbidities, and their parents/carers participated in a twice weekly, 10-week after-school child obesity treatment program between 2009 and 2012. Outcome information included measures of weight and weight-related behaviours. Analyses were adjusted for clustering and socio-demographic variables.

**Results:**

Overall, 2,812 children participated (54.2 % girls; M_age_ 10.1 (2.0) years; M_attaendance_ 12.9 (5.9) sessions). Beneficial changes among all children included BMI (−0.65 kg/m^2^), BMI-z-score (−0.11), waist circumference (−1.8 cm), and WtHtr (−0.02); self-esteem (+2.7units), physical activity (+1.2 days/week), screen time (−4.8 h/week), and unhealthy foods index (−2.4units) (all *p* < 0.001). Children who completed **≥**75 % of the program were more likely to have beneficial changes in BMI, self-esteem and diet (sugar sweetened beverages, lollies/chocolate, hot chips and takeaways) compared with children completing <75 % of the program.

**Conclusions:**

This is one of the few studies to report outcomes of a government-funded, program at scale in a real-world setting, and shows that investment in a community-based child obesity treatment program holds potential to produce short-term changes in weight and weight-related behaviours. The findings support government investment in this health priority area, and demonstrate that community-based models of child obesity treatment are a promising adjunctive intervention to health service provision at all levels of care.

## Background

One in four Australian children aged 5–14 years are overweight or obese [1]. This equates to 716,000 children (based on 2011 census data) and current Australian tertiary paediatric obesity services are unable to meet the treatment requirements of these children [2]. The evidence suggests that screening and brief counselling in primary care settings is not effective in reducing weight and improving weight-related behaviours in overweight/obese children and, that the costs associated with this strategy would be better spent on enhancing other treatment options [3]. Community-based obesity treatment programs have therefore become an important response to address child obesity; however the majority of these programs are small, efficacy trials [4].

In public health, once interventions have demonstrated efficacy through randomised controlled trial designs (RCT; i.e., innovation testing), the next steps are to translate the research through replication and to scale-up and disseminate the interventions to a much larger population in the ‘real-world’ setting [5]. RCTs are the gold standard of research designs, however they are typically conducted on narrowly defined populations under strict conditions with intensive intervention which are difficult to replicate in the real world. In terms of community-based obesity treatment programs, the external validity (i.e., generalizability to other populations and settings) and internal validity (i.e., change attributed to the intervention alone and not to an alternative factor) have received less attention, resulting in a major knowledge gap in our understanding of how such interventions may work in the real world [6–8].

MEND is a UK community-based child obesity treatment intervention [8–11] designed to be scalable and delivered by a range of health professionals. The program has been translated into the Australian context as Go4Fun® and assessing its effectiveness is critical to the success of examining comprehensive population approaches to childhood obesity treatment and prevention. For policy makers, information on whether scaled-up interventions are viable across a variety of communities and circumstances is important to ensure best practices for the community are adopted. The process evaluation and details of the Go4Fun® program sessions have been published [12]. The aim of this paper is to report the programs’ outcomes following scaling-up across New South Wales (NSW), Australia’s most populous state (pop: 7.2mil). We hypothesised that children’s weight and weight-related behaviours would improve in the desired direction and the strength of improvements would be stronger among children who participated in ≥75 % of the programs sessions.

## Methods

### Design

A pre-post non-controlled design was used to report the outcomes of the Go4Fun program implemented between 2009 and 2012. In total, 293 programs were delivered by 15 Local Health Districts (LHD) across NSW. Government funding for the program is based on the proportion of overweight/obese children and geographical spread of each LHD. Written consent by a parent was a requirement for participation. The study was approved by the University of Sydney Human Research Ethics Committee.

### Participants

Participants were either self-referrals (via a toll-free phone number, a text message or online registration) or referred by health professionals, relevant organisations and community members. Whilst the Ministry of Health centrally manages the program, each LHD undertakes recruitment within their area (via local media, health services, schools, councils and non-government organisations) and participates in local partnerships and promotions to target communities at social disadvantage. Children were eligible for the program if they were aged 7–13 years, however a small number of children whose birthdays were close to the age criteria were included in the program and the analysis (age 6.2-6.9 years *n* = 70 and 14.0-14.8 years *n* = 19). Children had to be overweight or obese (body mass index [BMI] ≥85th percentile based on the CDC growth references) [9], with no co-morbidities, and have a parent/adult carer to accompany them to each session. Eligibility was assessed at the time of referral/contact with LHDs and based on anthropometric measures and a medical history questionnaire completed by a parent/carer.

### Program description

Go4Fun is a replication of the MEND program and the details of the program format and components have been published [10, 11]. Briefly, MEND Australia Pty Ltd is responsible for providing the Go4Fun program to the NSW Ministry of Health, including delivery of program training, resources, tools, infrastructure to monitor and track participants through the program, and program fidelity. The program comprises 2-h sessions twice a week for 10 consecutive weeks (i.e., 20 sessions), after-school during school terms. The sessions address key components for individual-level behavioural change including education, skills training, and motivational enhancement [10]. At each LDH the sessions were conducted by the same facilitators who received two days of face-to-face and online training facilitated by MEND Australia.

Data on all children enrolled in 293 programs across the 15 LHDs between 27th July 2009 and 22nd October 2012 were examined. At enrolment, parents provided socio-demographic information including their child’s sex, date of birth, postcode of usual residence, and Aboriginal status. Postcode of residence was used as a proxy for socioeconomic status (SES), based on the Australian Bureau of Statistics’ Socio-Economic Indexes for Areas (SEIFA) [12] and the Accessibility-Remoteness Index of Australia Plus (ARIA+) which determines geographical remoteness [13]. SES was based on SEIFA quintiles and ARIA+ measures were categorised as major city and other (i.e., inner regional, outer regional, remote/very remote).

### Outcome measures

One trained facilitator at each LHD collected outcome measures at the first and last session of the program. Measures included the child’s height (m), weight (kg) and waist circumference (cm). BMI (kg/m^2^) was calculated and BMI z-scores determined from CDC reference data [9]. Waist-to-height ratios (WtHtr) were derived and categorised as <0.5 and ≥0.5 [14].

The UK MEND questionnaires and measures were used to assess differences between outcomes derived from a controlled trial situation and real world implementation. Parents completed the questionnaires on their child’s weight-related behaviours including the number of days a week their child spent engaging in one hour or more of moderate-intensity physical activity, and their perception of how active their child was compared with same-aged peers. Cardiovascular fitness was assessed by heart rate recovery one minute after completing a height-adjusted 3-min step test [15]. Screen-time was determined from the question “how many hours per week does your child spend watching TV/DVDs/video or playing on the computer/video games?”

Indicators of dietary habits and patterns included the frequency of eating breakfast (Response categories rarely; a few times a month; a few times a week; most days of the week and; everyday), daily serves of fruit and vegetables (open ended), consumption of sugar-sweetened beverages, potato chips, lollies/chocolate, and takeaway foods (response categories rarely; once a week; a few times a week; most days of the week and; everyday). For the analysis, the responses were dichotomised as ‘not frequent’ (rarely once a week) and ‘frequent’ (a few times a week; most days of the week and; everyday). An unhealthy food score was also derived as an index of the frequency of eating unhealthy foods (i.e., sugar-sweetened beverages, potato chips, lollies/chocolate, and takeaway foods), with higher scores indicating a higher frequency of eating unhealthy foods. Parents also reported on their comprehension of nutrition labels, how often they cooked “fresh food from scratch at home”, and how often the family ate meals together at the table. Children completed the adapted Rosenberg Self-Esteem Scale, which comprises 10 items on a four-point Likert scale, with higher values (score range: 0–30) indicating higher levels of self-esteem [16].

### Analysis

Data analysis was undertaken using SAS software (version 9.3, SAS system for Windows). The purpose of this study was to report change in real-world, uncontrolled circumstances so missing values were not imputed. However we did assess imputation of baseline values and the direction and order of magnitude of coefficients were similar in those estimated using complete case data. Additionally, to provide policy-makers with evidence based on real world compliance where the threat of not achieving 100 % participation in community-based programs must be considered we made a pragmatic decision which would be considered useful for policy decisions and defined ‘completers’ as children who attended ≥75 % of intended program sessions and non-completers as children who attended <75 % of the program.

For continuous variables mixed models were used to estimate the mean values and 95 % confidence intervals (CIs) at each time point (before and after the program) controlling for age, sex, season, area level of socio-economic disadvantage (SEIFA, quintiles), area level of remoteness (ARIA+), and the number of sessions attended. Participant was modelled as a random factor to account for the paired before and after values on the same participant. Programme ID was also modelled as a random factor to account for the cluster design of the study. The mean change in outcome between before program attendance and after the program was assessed using a linear mixed model with change as the dependent variable and controlling for baseline as well as the factors above but with only Programme ID as a random factor. Effect sizes were determined using Cohen's d [17] and calculated using mean differences and standard errors from the mixed model where 0–0.2 is small, 0.2-0.4 is medium and 0.4+ is a large effect size.

For categorical dietary variables, generalized linear mixed models were used to calculate the prevalence of being in the frequent consumption group before the program and after the program. Again the model was controlled for the relevant fixed and random effects. The prevalences were compared using odds ratios and their 95 % confidence intervals to compare the odds of being in the frequent consumption group after the program as compared with before.

Further modeling was done to compare the outcomes of completers and non-completers. The models were analogous to those previously described but instead of number of sessions included as in independent variable in the model, a binary variable of completers versus non completers was included. In the continuous case, change from baseline scores were modeled as the dependent variable and the mean change in each group of completers and non-completers was calculated and then compared. In the binary outcome case, the probability of being in a high consumption group was modeled as the dependent variable through a logit transformation. A completer versus time interaction was added to the models to calculate both the odds ratios for the odds of being in the high consumption group after the program compared with before for the completers and non-completers groups separately. The overall odds ratios comparing completers and non-completers over both time points were also calculated. The linear mixed models were implemented in SAS v9.3 using proc mixed for the continuous outcomes and PROC GLIMMIX for the binary outcomes. Model parameters were estimated using residual maximum likelihood and residual pseudo-likelihood methods respectively.

## Results

Between 27th July 2009 and 22nd October 2012, 3,148 overweight/obese children age 6–15 years (girls = 54.2 %; M = 10.1; SD = 2.0) were recruited in 293 programs in 15 LHD’s. Of these children, 336 (10.7 %) did not attend any sessions 2,812 attended one or more sessions (89.3 % median number of participants per programme =9, range 1–20). Of the 2,812 children who attended the program, 1,520(54.1 %; median number of participants per programme =5, range 1–13) completed ≥ 75 % of sessions (‘completers’) with 56.0 % (*n* = 851) attending ≥ 90 % of sessions. Completers were more likely to be girls (*p* = .008), non-Aboriginal (*p* = .001), and to live in less socially disadvantaged areas (*p* < .001) (Table 1). There were no differences between completers and non-completers anthropometry.

**Table 1:** Baseline demographic and anthropometric characteristics all children participating in at least 1 session, attending ≥75 % (completers) and <75 % (non-completers) of the program, 2009-2012

	Program attendance			
Characteristics	All participants	Completers	Non-completers	P-value*
n	2,812	1,520 (54.1 %)	1,292(46.0 %)	
Mean sessions attended (n; SD)	12.9 (5.9)	17.5 (1.8)	7.6 (4.3)	
Girls (%)	54.2	56.5	51.5	.008
Boys (%)	45.8	43.5	48.5	
Age (years; mean (SD))	10.1 (2.0)	10.1 (1.8)	10.1 (2.1)	.383
Weight status (%)				
Overweight	28.2	28.3	28.1	.909
Obese	71.8	71.7	71.9	
BMI z-score (mean [SD])	2.08 (0.41)	2.07 (.40)	2.09 (.42)	.139
WtHtr ≥ 0.5 (%)	93.8	94.5	92.8	.064
Aboriginality (%)				
Non Aboriginal	94.6	95.9	93.1	.001
Aboriginal or Torres Strait Islander	5.4	4.1	6.9	
Residence (ARIA+) (%)				
Major city	63.1	61.7	64.7	.104
Other	36.9	38.3	35.3	
Socio-economic status (SEIFA index) (%)				
1st-2nd quintile (most advantaged)	22.4	25.3	18.8	<0.001
3rd-5th quintile (most disadvantaged)	77.7	74.7	81.2	

*ARIA+* Accessibility Remoteness Index of Australia Plus, *BMI* body mass index, *SD* standard deviation, *SEIFA* Socio-Economic Index for Areas, *WtHtr* waist-to-height ratio

*P-value difference between completers and non-completers

**Fig. 1:**
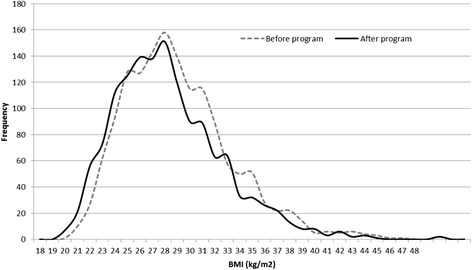
BMI distribution curves of completers (attended ≥75 % of sessions) at baseline and program completion (*n* = 1,520)

Table 2 shows the mean changes and odds ratios in program outcomes, adjusting for covariates (*n* = 2,812; mean attendance = 12.9 sessions). Overall, the mean changes in program outcomes were statistically significant and in the desired direction (all *p* < .001), with the exception of understanding nutrition labels, which showed a 77 % reduction in ‘always’ understanding nutrition labels after attending the program (OR 0.23; 95 % CI 0.20, 0.26). Medium effects sizes (d = .20-.38) were observed for change before and after the program in self-esteem, screen time, physical activity, daily vegetable intake, and a large effect size for change in unhealthy food index (d = .41). Tables 3 and 4 show the mean change in program outcomes according to program attendance (i.e., non-completers vs completers). Compared with non-completers, greater beneficial changes were observed among children who completed the program for BMI, BMI z-score, unhealthy food index, and the frequency of consuming sugar sweetened beverages, lollies/chocolates, potato chips and takeaways. Figure 1 graphically shows the left shift in the BMI distribution curves of completers before and after participating in the program (*p* **<** .001).

**Table 2:** Mean changes in program outcomes for all child participants (*n* = 2,812)^a^

All child participants	n^b^	Before program	After program	Mean change	P-value	Effect size
		Mean (95 % CI)	Mean (95 % CI)	(95 % CI)		(Cohen’s d)
BMI (kg/m^2^)	1,782	26.7 (26.3, 27.1)	26.0 (25.6, 26.4)	−0.65 (−0.78, −0.53)	*<.001*	−.07
BMI z-score	1,776	2.07 (2.04, 2.11)	1.96 (1.93, 2.00)	−0.11 (−0.12, −0.09)	*<.001*	−.13
Waist circumference (cm)	1,769	86.6 (85.5, 87.8)	84.2 (83.1, 85.4)	−1.83 (−2.61, −1.05)	*<.001*	−.09
Waist-to-height ratio	1,769	0.60 (0.59, 0.61)	0.58 (0.57, 0.58)	−0.02 (−0.02, −0.01)	*<.001*	−.12
Global self-esteem score (range: 0–30)	1,348	17.9 (17.2, 18.5)	20.7 (20.1, 21.3)	2.73 (2.06, 3.41)	*<.001*	*.20*
Screen-time^c^ (hours/week)	1,328	13.0 (12.3, 13.8)	8.28 (7.47, 9.08)	−4.78 (−5.53, −4.03)	*<.001*	*−.27*
Recovery heart rate (beats/min)	1,733	109.7(107.1, 112.3)	102.5 (99.9, 105.1)	−4.64 (−7.57, −1.72)	*0.002*	−.11
Physical activity ≥1 h/session (days/week)	1,348	1.73 (1.56, 1.89)	2.91 (2.74, 3.08)	1.24 (1.01, 1.47)	*<.001*	*.31*
Daily serves of fruit (n)	607	1.90 (1.67, 2.13)	2.25 (2.01, 2.49)	0.16(−0.12, 0.45)	*<.001*	.10
Daily serves of vegetable (n)	582	1.92 (1.63, 2.20)	2.55 (2.26, 2.84)	0.78 (0.35, 1.23)	*<.001*	*.38*
Unhealthy food index^d^ (score range: 0–20)	1,549	7.64 (7.35, 7.94)	4.70 (4.39, 5.01)	−2.42 (−2.82, −2.02)	*<.001*	.*41*
Probability of consuming item ‘frequently’		% (95 % CI)	% (95 % CI)	OR (95 % CI)		
Sugar-sweetened beverages (%)	1,598	0.73 (0.70, 0.78)	0.56 (0.55, 0.58)	0.25 (0.21, 0.30)	<.001	−
Lollies/chocolate (%)	1,598	0.86 (0.81, 0.89)	0.59 (0.57, 0.60)	0.20 (0.17, 0.23)	<.001	−
Potato chips (%)	1,597	0.79 (0.75, 0.83)	0.56 (0.55, 0.57)	0.19 (0.16, 0.22)	<.001	−
Takeaways (%)	1,536	0.57 (0.55, 0.58)	0.51 (0.51, 0.52)	0.17 (0.13, 0.22)	<.001	−
Daily breakfast (%)	1,508	0.60 (0.58, 0.62)	0.99 (0.99, 1.00)	12.83 (10.87, 15.14)	<.001	−
Probability of always/often undertaking behaviour						−
Often/always understand nutrition labels (%)	1,535	0.99 (0.97, 0.99)	0.73 (0.69, 0.77)	0.23 (0.20, 0.26)	<.001	−
Often/always cooking fresh food from scratch (%)	1,537	0.59 (0.57, 0.61)	0.64 (0.62, 0.67)	1.59 (1.37, 1.83)	<.001	−
Often/always eating dinner as a family (%)	1,539	0.61 (0.59, 0.64)	0.65 (0.62, 0.68)	1.30 (1.12, 1.50)	<.001	−

*BMI* body mass index, *CI* confidence intervals, *SD* standard deviation

^a^Adjusted for baseline values, age, sex, SEIFA, ARIA+; season, and the number of program sessions attended

^b^Numbers vary due to missing data

^c^Watching TV/DVDs/videos or playing electronic/video games

^d^Based on frequency of consumption of unhealthy foods (i.e., potato chips, lollies, chocolate, takeaway foods and sugar-sweetened soft drinks); higher scores represent a less healthy food index

**Table 3:** Mean change in outcomes according to program attendance; <75 % (*n* = 1,164) and ≥ 75 % (*n* = 1,486)^a^

	Mean change (95 % CI) by attendance		Mean difference (95 % CI)	P value	Effect size (Cohen’s d)
	Non-completers	Completers			
BMI (kg/m2)	−0.49 (−0.64, −0.34)	−0.70 (−0.83, −0.57)	−0.20 (−0.31, −0.10)	<.001	−.09
BMI z-score	−0.08 (−0.10, −0.06)	−0.11 (−0.13, −0.10)	−0.03 (−0.02, −0.04)	<.001	−.10
Waist circumference (cm)	2.00 (1.09, 2.91)	1.79 (1.01, 2.58)	−0.21 (−0.80, 0.38)	.490	−.01
Waist-to-height ratio	−0.02 (−0.02, −0.01)	−0.02 (−0.02, −0.01)	0.000 (−0.004, 0.003)	.960	−.001
Global self-esteem score (range: 0–30)	−2.23 (−3.12, −1.33)	−2.84 (−3.52, −2.16)	−0.61 (−1.32, 0.10)	.093	−.06
Screen-time^b^ (hours/week)	−5.11 (−6.09, −4.13)	−4.71 (−5.47, −3.95)	0.40 (−0.37, 1.17)	.312	.03
Recovery heart rate (beats/min)	−3.92 (−7.12, −0.73)	−4.81 (−7.75, −1.87)	−0.88 (−2.50, 0.74)	.285	−.02
Physical activity ≥1 h/session (days/week)	1.17 (0.88, 1.47)	1.26 (1.02, 1.49)	0.08 (−0.011, 0.31)	.329	*−.25*
Daily serves of fruit (n)	0.25 (−0.09, 0.58)	0.15 (−0.13, 0.44)	−0.09 (−0.29, 0.11)	.370	−.03
Daily serves of vegetable (n)	0.74 (0.24, 1.24)	0.80 (0.36, 1.24)	0.06 (−0.20, 0.32)	.671	.01
Unhealthy food index^c^ (range: 0–20)	−2.05 (−2.55, −1.55)	−2.51 (−2.91, −2.10)	−0.45 (−0.82, 0.09)	.014	−.07

*BMI* body mass index, *CI* confidence intervals, *OR* odds ratio

^a^Adjusted for baseline values, sex, SEIFA, ARIA+; and season

^b^Watching TV/DVDs/videos or playing electronic/video games

^c^Based on frequency of consumption of unhealthy foods (i.e., potato chips, lollies, chocolate, takeaway foods and sugar-sweetened soft drinks); higher scores represent a less healthy food index

**Table 4:** Mean change in Odds Ratios (OR) in program outcomes for non-completers and completers^a^

	Odds after programme compared with before programme		Odds (Completers compared with non-completers overall) (95 % CI)	P value
	Non-completers	Completers		
Probability of consuming food/beverage item ‘frequently’	OR (95 % CI)	OR (95 % CI)	OR (95 % CI)	
Sugar-sweetened beverages	0.23 (0.17, 0.47)	0.25 (0.21, 0.31)	0.70 (0.57, 0.85)	<.001
Lollies/chocolate	0.20 (0.16, 0.26)	0.20 (0.16, 0.23)	0.76 (0.64, 0.90)	.002
Potato chips	0.19 (0.14, 0.25)	0.18 (0.15, 0.22)	0.69 (0.57, 0.84)	<.001
Takeaways	0.17 (0.10, 0.28)	0.15 (0.11, 0.21)	0.72 (0.53, 0.98)	.038
Daily breakfast	5.68 (4.26, 7.58)	16.7 (13.7, 20.4)	1.15 (0.96, 1.36)	.126
Probability of always/often undertaking behaviour				
Often/always understand nutrition labels	0.23 (0.13, 0.17)	0.26 (0.22, 0.31)	1.06 (0.90, 1.25)	.480
Often/always cooking fresh food from scratch	1.48 (1.11, 1.96)	1.66 (1.40, 1.96)	0.96 (0.80, 1.16)	.687
Often/always eating dinner as a family	1.58 (1.18, 2.11)	1.24 (1.05, 1.47)	1.03 (0.85, 1.25)	.768

^a^Adjusted for baseline values, sex, SEIFA, ARIA+; and season

## Discussion

The purpose of this study was to report the outcomes of a known, effective child obesity treatment program (MEND) that was government-funded, scaled-up and implemented across communities in NSW, Australia. The findings indicate that in the short-term significant, albeit clinically small, improvements are achievable in children’s adiposity, weight-related behaviours. While there are no similar studies to compare with our findings, they are commensurate with those reported by the UK MEND at 6-months under an RCT design [11]. The improvements in BMI outcomes are approximately half those reported in the original efficacy trial, and although BMI z-score reductions ≥0.25 are associated with improved metabolic health in obese children [18], the left shift in the BMI distribution curve after the program is promising.

Differences in the magnitude of change and a higher retention rate in the original UK efficacy trial [11] may be due to the study design and dose. MEND was conducted as a small RCT (*n* = 116) while Go4Fun® has been scaled-up and disseminated into a real-world setting. Further, only obese children were enrolled in the UK program and the intervention was longer (9-week group program followed by a 12-week free family swimming pass, which incidentally had a poor uptake) [11].

Children who participated in Go4Fun did improve their screen-time, physical activity and, dietary habits; key weight-related behaviours which must precede change in adiposity. Importantly, improvements in children’s self-esteem suggest that attending the program had no negative effects on the children. An unexpected finding was the decline in parents understanding of nutrition labels on completion of the program. This may reflect the current confusing status in Australia of different nutrition labelling formats (e.g., %RDIs, star rating, Pick the Ticks, traffic lights etc.) [19] on food products and suggests that policy makers need to ensure nutrition labels are understandable by community members [20].

We examined outcome differences between completers and non-completers to inform policy decisions on the program’s delivery. Greater beneficial changes in BMI and diet behaviours were observed among children who completed the program compared with children who did not complete the program. Although children who completed the program did not consistently demonstrate better outcomes on all weight-related behaviours, the fact that children who completed the program had a significantly higher probability of decreasing their intake of ‘extra’ foods’ (i.e., junk food) is highly relevant given the evidence shows that Australian children over-consume such foods [21, 22]. Other research shows that parents believe that as long as children also eat ‘healthy’ foods then the frequent consumption of extra foods is acceptable [23]. Given the majority of Go4Fun sessions focused on diet (13/20), and completers attended almost 10 more sessions than non-completers this potentially indicates that improvements in food literacy require maximal program exposure in order for poor dietary behaviours to change.

Information on how scaled-up programs such as Go4Fun® perform in the real-world outside of controlled circumstances is limited and, yet for policy makers this information is critical for program funding and delivery decisions. Process evaluation of the program [24] showed that it reached predominantly obese children (70 % obese) and children from social disadvantaged backgrounds, but boys and Aboriginal children were less likely to complete the program. Accordingly, a specific, more culturally appropriate program is currently being developed for Aboriginal children and families and will involve an RCT to ascertain its efficacy. Similarly, qualitative work (e.g., focus groups) will help identify strategies to retain boys in the program.

There are a number of limitations to consider in the interpretation of our findings. The high rate of missing data has the potential to introduce bias which may impact on the effect sizes reported here. Parents were asked to complete a questionnaire on behalf of their child at the first and last session, with the Go4Fun facilitators, yet for unknown reasons, many questions were not completed suggesting our data were missing at random. Both the questionnaire and software were developed by MEND Australia, however, there were issues with data entry (i.e., no data entry controls) and the high rate of missing data suggests better facilitator training is required to ensure program fidelity in real-world situations. Similarly the reliability the anthropometric measurements was not assessed so the level of measurement error is unknown. Children’s pubertal status was not measured because of the invasiveness of such tests in a community-based program and may have confounded our results given maturation has a large independent impact on adiposity, fitness and cardio-metabolic risk.

The lack of long term follow-up is an obvious and significant limitation as these data would provide valuable information on the sustainability of positive change in weight-related behaviours, which would further inform program planning. There is no definition of completion for community based interventions so defining completion as ≥75 % attendance could be considered contentious, however 100 % attendance is an unrealistic expectation when shifting from controlled to real-world implementation. Attrition rates from paediatric weight-loss programs are not known, but among adults attrition rates vary from 10 to 80 % with the most predictive correlates for dropping out being high treatment expectations and low self-efficacy [25]. For policy makers, findings on children who complete the majority (i.e., ≥75 %) of a public health program such as Go4Fun provides realistic information about expected outcomes when translating efficacy trials into the broader community.

The finding that improvements in weight and weight-related outcomes remained significant after controlling for potential confounders suggests that the program helped facilitate positive lifestyle changes in the children. Furthermore, almost three-in-five children completed the program which suggests that government-funded, community-based child obesity treatment programs do resonate with most participants, can be scaled-up and provide improvements in weight and behavioural outcomes [26]. Longer-term follow-up of participants is essential to ensure that changes in weight-related behaviour adopted during a short-term intervention are sustainable. Go4Fun provides a starting point for realising the potential of scaling-up small efficacy trials into the real-world; the next and necessary steps in translational research in a health priority area.

## Conclusions

Go4Fun® programs demonstrated small but beneficial changes in overweight and obese children’s weight and weight-related behaviours in the short-term. Completing the program was associated with greater improvements in diet, but not other weight-related behaviours. An important policy decision which will influence funding is to examine the current program format to determine the optimal number of sessions required to achieve desired outcomes. The outcomes provide promise that community-based child obesity treatment programs are an adjunctive intervention to tertiary obesity health service, but long term follow up is necessary. Go4Fun is a service delivery program, however strategies to minimise missing data would assist the ongoing evaluation process.
